# Increased Serum AMH in a Subgroup of Women with Idiopathic Hyperandrogenism: Do These Women Have PCOS?

**DOI:** 10.3390/biomedicines13081942

**Published:** 2025-08-08

**Authors:** Enrico Carmina, Rogerio A. Lobo

**Affiliations:** 1 Endocrinology Unit, School of Medicine, University of Palermo, 90139 Palermo, Italy; 2 Department of Obstetrics and Gynecology, Columbia University, New York, NY 10032, USA; ral35@cumc.columbia.edu

**Keywords:** AMH, PCOS, idiopathic hyperandrogenism, PCOS phenotypes

## Abstract

**Background:** Idiopathic Hyperandrogenism (IH) is often encountered in the general population and particularly in women presenting with androgen excess. It is diagnosed in women who have normal ovulatory cycles, elevated androgen levels, and normal appearing ovaries on ultrasound. Using serum anti-mullerian hormone (AMH), which has been suggested to be a surrogate marker of polycystic ovarian morphology, we wished to determine if some women with IH may have the ovulatory phenotype of PCOS, where metabolic abnormalities may be more prevalent. **Methods:** This was a retrospective study of 84 women diagnosed with IH and 50 age- and BMI-matched ovulatory controls who were evaluated between 2021 and 2024. Androgen levels, ovarian ultrasound, and serum AMH were assessed. In addition, glucose, insulin, HOMA-IR, and lipid levels were measured. **Results:** Twenty-four women (29%) with IH had elevated AMH levels (>4.7 ng/mL). None of the control women had elevated values. These 24 women with IH had values ranging from 5.5–11.9 ng/mL, compared to the other 60 women whose AMH levels were 0.8–4.3 ng/mL. Age and weight were similar in these two subgroups of women with IH. Glucose, insulin, and HOMA-IR were similar in the two groups, but cholesterol and LDL-cholesterol were elevated in the 24 women with elevated AMH. Elevations in LDL-cholesterol were observed in 3.5% of the women with normal AMH and in 21% of those with elevated AMH. **Conclusions:** Despite normal ovarian morphology on ultrasound, approximately 30% of women with IH were found to have elevated AMH and may be considered to have the ovulatory phenotype of PCOS. These women also have some metabolic dysfunction, which is more characteristic of women with PCOS. These data reinforce the notion that AMH findings are not always concordant with ovarian morphological findings and suggest wider implication of elevated AMH levels in the pathophysiology of PCOS.

## 1. Introduction

Idiopathic Hyperandrogenism (IH) is a disorder that is not uncommonly found in the general population [1,2] but is less prevalent than Polycystic Ovary Syndrome (PCOS) in women presenting with symptoms of androgen excess [3]. It is diagnosed by the finding of elevated androgen levels in blood in normal ovulatory women who do not demonstrate polycystic ovarian morphology on ultrasound [1,2,3,4]. It is differentiated from the ovulatory phenotype of Polycystic Ovary Syndrome (PCOS) (phenotype C) by women with IH not having ultrasonographic evidence of polycystic ovaries [4].

Here we postulated that some women diagnosed to have IH, may indeed be considered to have the ovulatory phenotype of PCOS. Identification of such cases may be important because women with PCOS, even women with phenotype C, frequently have abnormal metabolic features [5], which are absent in women with IH [4]. In women with PCOS, Anti-Mullerian Hormone (AMH) levels in blood are increased and correlate positively with ovarian follicular counts, with androgen levels, as well as with the severity of the symptoms of PCOS [6,7,8,9,10,11,12]. Although the role of serum AMH in the diagnosis of PCOS remains controversial, recent expert guidelines have suggested that serum AMH may be used as a substitute for the finding of polycystic ovaries on ultrasound and used as one of the criteria for the diagnosis of PCOS [13]. One of the controversies regarding the use of serum AMH in the diagnosis of PCOS is that it lacks sensitivity. It is generally less sensitive than follicle number per ovary and is less sensitive in certain phenotypes, such as phenotypes C and the non-androgenic phenotype of PCOS (phenotype D) [12,14]. However, when levels of AMH are significantly elevated, it suggests a more severe state of ovarian dysregulation, as observed in phenotype A [12,14]. In this study, our objective was to examine whether there are alterations in serum AMH in women diagnosed with IH who by definition have “normal” ovarian morphology. Our hypothesis was that AMH levels may be elevated in some women diagnosed with IH. Indeed, as reported here, we found that a subgroup of patients with IH have elevated levels of serum AMH and may be considered to have a form of PCOS.

## 2. Materials and Methods

Eighty-four women (mean age 24 ± 3 years, range 19–29 years) who were referred to the Endocrine Clinic, University of Palermo, Palermo, Italy, between 2021 and 2024 because of clinical and biochemical hyperandrogenism and who were diagnosed to have Idiopathic Hyperandrogenism (IH) were studied retrospectively. In all patients, the diagnosis of IH was based on the findings of increased serum androgens associated with hirsutism and/or adult acne with normal ovulatory menstrual cycles and normal ovaries as assessed by ovarian sonography [4]. All subjects underwent a complete history and physical examination, biochemical analyses, and transvaginal ultrasonography using an 8–10 MHz transducer.

Fifty normal ovulatory women were selected as controls. These women were drawn from the same population base, had no complaints related to androgen excess, and were matched for age (mean age of 24 ± 3 years, ranged 18–29 years) with our patient population of women with IH. Mean BMI was normal and similar in patients with IH versus normal controls (mean BMI 23.3 ± 3 versus 23.6 ± 3 kg/m^2^). The controls underwent the same endocrine and metabolic evaluations and ultrasound evaluations as women diagnosed with IH. If any abnormalities were encountered, according to our protocol, these women would have been excluded from the control group, but this did not occur for any subject recruited in this study.

All patients seen consecutively between 2021 and 2024 who were diagnosed with IH were entered into the analysis. A large group of 84 women were included. Since we did not know how many women would have or not have elevations in AMH, there was no possibility of carrying out formal power calculations. For this number of women with IH, 50 matched ovulatory controls were considered adequate for comparisons.

Although the study analyses were carried out retrospectively, all patients were contacted and given informed consent for this evaluation. Our research protocol was approved by our Institutional Review Board (Approval Code: 2018/23, Approval Date: 10 July 2018).

### 2.1. Laboratory Analyses

In all patients and controls, serum levels of total testosterone (T), androstenedione (A), dehydroepiandrosterone sulfate (DHEAS), and AMH were measured on days 3–5 of the cycle. In all patients, serum 17-hydroxyprogesterone (17OHP) values were measured to exclude the existence of Non-Classic Congenital Adrenal Hyperplasia [15]. All controls and patients had serum progesterone measured on days 21–22 of the cycle to confirm the occurrence of ovulation. In all women with IH and controls, a metabolic profile was obtained. The metabolic profile included measurements of fasting blood levels of glucose, insulin, total cholesterol, high-density lipoprotein cholesterol (HDL-C), low-density lipoprotein cholesterol (LDL-C), and triglycerides (TG). Insulin sensitivity was evaluated by the quantitative HOMA-IR method [15]. Total T was determined by mass spectrometry after liquid chromatography (LC/MS) assay [15], while the other steroid hormones were measured by specific RIAs using previously described methods [16]. Biochemical hyperandrogenism was defined as serum T > 34 ng/dL and/or serum A > 2.9 ng/mL and/or serum DHEAS > 3 µg/mL. These values for hyperandrogenism have been previously validated with the use of the previously described assays [16]. For AMH measurements, samples were collected into serum tubes with gel separators and centrifuged within 5 h. AMH was measured using a highly specific assay as previously described [12], and values >4.7 ng/mL were considered elevated [12].

In all assays, intra-assay and inter-assay coefficients of variation did not exceed 6% and 15%, respectively.

### 2.2. Ovarian Ultrasound

Transvaginal pelvic ultrasound was performed using a transducer frequency of 8–10 MHz, and the presence of polycystic ovaries was established by the finding of increased numbers of follicles, each of which measured 2–10 mm in diameter, and/or increased ovarian size [17]. According to our previous study [12], the threshold for increased follicle number per ovary (FNPO) was 22 follicles per ovary. All patients diagnosed with IH and all controls had follicle numbers per ovary (FNPO) under this threshold of 22. No patient had received any medication for at least 3 months before the study.

### 2.3. Statistics

Statistical analyses were performed using Statview 5.0 (SAS Institute, USA). Since several values were not normally distributed, a log transformation was necessary to obtain a normal distribution. Mann–Whitney tests were performed to compare parameters between women with IH and controls. Cut-off values for elevations compared to the control population were determined using ROC curve analysis. Here, a plot of sensitivity against 1-specificity provides the curve analysis. The area under the curve of this plot provides statistical information about sensitivity and specificity for various threshold values. ANOVA followed by the Tukey test was performed to assess the differences in clinical and biochemical parameters between different subgroups of women with IH. A *p* value < 0.05 was regarded as statistically significant.

## 3. Results

Compared to controls, women diagnosed with IH had significantly (*p* < 0.01) elevated levels of serum AMH, testosterone, androstenedione, and DHEAS (Table 1). Twenty-four women with Idiopathic Hyperandrogenism (29%) had increased AMH values (mean AMH ± SD 7.3 ± 2.3 ng/mL, range 5.5–11.9 ng/mL), while 60 patients with IH had normal AMH values (mean AMH ± SD 2.7 ± 1.3 ng/mL, range 0.8–4.3 ng/mL). The two groups of patients with IH were of similar age (24.6 ± 3.3 versus 24.6 ± 3.5 years) and BMI (23.6 ± 3 versus 23.4 ± 4), but the IH patients who had increased values of AMH had higher levels of total testosterone, androstenedione, and DHEAS compared to those IH patients with normal AMH values (Table 2). The two groups of women with IH had similar levels of fasting serum glucose, insulin, HDL-cholesterol, and triglycerides. However, serum total and LDL-cholesterol values were significantly (*p* < 0.05) higher in IH patients with high AMH values compared to those with normal AMH values (Table 3). On an individual basis, two women with IH and normal AMH values (3.5%) had elevated LDL-cholesterol values, while five (21%) of those women with elevated AMH values had elevated LDL-cholesterol.

## 4. Discussion

In this study, we report that approximately 30% of women with a diagnosis of IH who by definition have normal ovaries on ovarian sonography have elevated values of AMH. Previously, we reported that in women with PCOS, AMH is increased and correlates with androgen levels, ovarian size, and insulin resistance [6]. Many studies have confirmed the elevation of AMH in women with PCOS and have shown that circulating levels correlate positively with follicular number per ovary (FNPO) and with androgen levels as well as the severity of the symptoms of PCOS [6,7,8,9,10,11]. Recently, a group of experts providing consensus guidelines regarding PCOS included serum AMH as a diagnostic tool for PCOS and suggested that the measurement of AMH may serve as an alternative to the ovarian sonographic finding of polycystic ovaries [13]. However, while AMH is the product of granulosa cells of small preantral follicles, which are increased in women with polycystic ovaries and have a role in inhibiting FSH action, contributing to anovulation [18,19,20], AMH levels are not elevated in all cases of PCOS [21]. It has been reported that up to 50% of women with ovulatory PCOS (phenotype C) have normal AMH values [12,14]. The reverse may also occur, where women with phenotype B (menstrual irregularity and hyperandrogenism but with normal ovaries) may have elevations in AMH [12]. Thus, it appears that while AMH usually reflects polycystic ovarian morphology, there are other aspects of AMH which are important in the pathophysiology of PCOS.

Genetic and epigenetic studies have suggested a much more important role for AMH in the pathogenesis of PCOS than just reflecting the number of follicles in the ovaries [22]. An altered gene variant has recently been reported in a large GWAS study of women with PCOS [23]. Rare variants of the AMH gene have been reported and may be implicated in a smaller number (up to 10%) of cases of PCOS [24]. In addition, the possibility that increased AMH values in pregnant women may cause epigenetic alterations in AMH production in the fetus has been clearly demonstrated [18,25,26]. All these data suggest that increased AMH values are not simply a reflection of increased follicle numbers in the ovaries but may reflect a significant alteration in ovarian function, perhaps even in the absence of polycystic ovarian morphology on ultrasound.

As stated above, we report here that approximately 30% of women with a diagnosis of IH who by definition have normal ovaries on ovarian sonography have elevated values of AMH. All women identified here had elevated levels of AMH, similar to cut-off values used for the diagnosis of PCOS. Interestingly, this subgroup of women, compared to women with the same diagnosis, had higher androgen values and also more elevated values of total cholesterol and LDL-cholesterol. These observations were not influenced by differences in BMI, which were similar. The finding of elevated LDL-cholesterol in the subgroup of women with IH is unique and important, but because of a small sample size, we cannot exclude the possibility of a type 1 statistical error.

IH is a disorder that has received relatively little attention and may be confused with women considered to have Idiopathic hirsutism [4,16,27]. However, patients with Idiopathic Hyperandrogenism (IH) are unique in that they have clearly elevated levels of serum androgens (mainly androstenedione and testosterone), and IH may be found in up to 10% of women being assessed for hirsutism [3,4,16]. Here, we show that a subgroup of women with a diagnosis of IH (up to 30%) are similar to those with ovulatory PCOS phenotype C in that they have elevated levels of AMH. Traditionally, women with phenotype C have ultrasound criteria for the diagnosis of PCOS. If we adapt the international guidelines where AMH may be used in lieu of ovarian ultrasound, approximately 30% of women with IH who were studied here indeed have PCOS.

Furthermore, the importance of these observations is that metabolic dysfunction in these women is higher compared to women with IH who have normal AMH values. Although not uniformly confirmed, there are studies suggesting that elevated AMH correlates with increased metabolic dysfunction [28,29] Note that the metabolic disturbance is also high in women with PCOS phenotype B (hyperandrogenism, anovulation, and normal ovaries), where it has been found that these women have elevated levels of AMH as well. More studies are needed to understand the lack of correlation between AMH and ovarian morphology in some cases, and perhaps the independent role of AMH elevation in the pathophysiology of PCOS.

The strength of this study is in the detailed evaluation of all women with IH including their ovulatory function, androgen elevations, and metabolic parameters, as compared to matched controls. The women in this study all had similar BMI and were young, having a narrow age range, which can also influence AMH levels. Furthermore, this study is fairly large, including 84 women with the diagnosis of IH.

The weakness of this study is that it is retrospective in nature. Women diagnosed with IH in our clinic were further assessed, with their consent, for features of PCOS as noted above. Ovarian sonography has also evolved over time and although this study was conducted fairly recently, it is possible that more detailed assessments of ovarian ultrasound may have provided greater clarity on this issue. As in all clinical studies, an even greater study size would have been preferable.

In summary, in this report, we provide greater insight into the disorder known as IH. Indeed, based on AMH values, up to 30% of these women may be considered to have PCOS. These women also have a more significant form of hyperandrogenism and exhibit some of the metabolic dysfunction characteristics of many women with PCOS. We suggest that it may be useful to carefully assess AMH levels in all women diagnosed with IH.

## Figures and Tables

**Table 1:** Mean (± SD) BMI and blood values of AMH and androgens in 84 patients with Idiopathic Hyperandrogenism.

	BMI Kg/m^2^	AMH ng/mL	Total Testosterone (T) ng/dL	Androstenedi one (A) ng/mL	Dehyroepiandro Sterone-Sulphate (DHEAS) μg/mL
Idiopathic Hyperandrogenism *n* = 84	23.6 ± 3	4.1 ± 2.7 *	39 ± 10 *	3.4 ± 1.2 *	2.8 ± 1.3 *
Normal controls *n* = 50	23.3 ± 3	2.9 ± 0.8	20 ± 6	1.8 ± 0.5	1.9 ± 0.5

* *p* < 0.01 for AMH, total testosterone, androstenedione and DHEAS versus controls.

**Table 2:** Mean (± SD values) serum androgen values in two subgroups of patients with Idiopathic Hyperandrogenism.

	AMH ng/mL	Total Testosterone (T) ng/dL	Androstenedione (A) ng/mL	Dehyroepiandroster One-Sulfate (DHEAS) μg/mL
Patients with increased AMH values *n* = 24	7.3 ± 2.3 **	46 ± 12 *	4.4 ± 0.7 **	3.5 ± 1.2 **
Patients with normal AMH values *n* = 60	2.7 ± 1.3	36 ± 7	3.2 ± 1	2.4 ± 0.6

* *p* < 0.05 for total testosterone; ** *p* < 0.01. for AMH, androstenedione and DHEAS.

**Table 3:** Mean (± SD values) circulating metabolic parameters in two subgroups of patients with Idiopathic Hyperandrogenism.

	Glucose mg/dL	Insulin mU/mL	Total Cholesterol mg/dL	HDL-Cholesterol mg/dL	Triglycerides mg/dL	LDL-Cholesterol mg/dL
Patients with increased AMH values *n* = 24	83 ± 9	9.6 ± 5	179 ± 32 *	57 ± 4	76 ± 22	107 ± 32 *
Patients with normal AMH *n* = 60	81 ± 8	9.3 ± 4	162 ± 30	57 ± 13	82 ± 38	89 ± 19

* *p* < 0.05 for total cholesterol and LDL-cholesterol; patients versus controls.

## Data Availability

Data supporting results can be found at the Endocrinology Unit of School of Medicine of University of Palermo.
